# Acute Toxic Cerebellar Leukoencephalopathy in an Eight-Year-Old Child Following Illicit Fentanyl and Cocaine Ingestion: A Case Report of Full Clinical Recovery

**DOI:** 10.7759/cureus.66573

**Published:** 2024-08-10

**Authors:** Alex M Gale, Daniel Nachreiner, Atul Kumar, Peter Sell, Stefanie Gauguet

**Affiliations:** 1 Pediatrics, UMass Memorial Medical Center, Worcester, USA; 2 Radiology, UMass Memorial Medical Center, Worcester, USA

**Keywords:** malignant cerebellar edema, somnolence, pediatric ingestion, fentanyl ingestion, acute toxic cerebellar leukoencephalopathy

## Abstract

During the current opioid epidemic, the number of children with illicit toxic ingestions is increasing. Children presenting with altered mental status and neurologic, particularly cerebellar symptoms of unclear etiology, should be considered to undergo brain imaging as well as toxicology screening to not miss the possible complication of acute toxic leukoencephalopathy.

We report the case of an eight-year-old child who presented with somnolence and respiratory depression of unclear etiology, responding profoundly to naloxone, quickly raising concern for drug ingestion. The toxicology screen was positive for fentanyl, cocaine metabolites, caffeine, and diphenhydramine, but not available until day 3 of the hospital stay. In the interim, head CT and brain MRI findings revealed concerning bilateral cerebellar hypodensities, suggestive of opioid-induced leukoencephalopathy. This condition has been described as potentially malignant and fatal, but very few cases of this pathology have been described in children so far. Fortunately, all neurological symptoms in our patient, including altered mental status, respiratory depression, atactic gait, blurry vision, and lower extremity pain, completely resolved within five days of presentation and the patient seemingly underwent a full clinical recovery without residual symptoms.

Awareness and prompt recognition of acute toxic leukoencephalopathy in children presenting with altered mental status or neurological symptoms of unclear etiology is of utmost importance to prevent deterioration and optimize treatment, especially during times of a worsening opioid epidemic in our country.

## Introduction

The United States has faced a nationwide substance abuse epidemic for the past 20 years leading to many overdose-associated deaths, with the majority (60%) being due to opioids [1-3]. Fentanyl is now the most common opioid leading to substantially increasing numbers of pediatric deaths [4].

Opioid overdoses can cause respiratory depression, as well as multi-organ system failure due to hypoperfusion and hypoxia. Opioids can also have neurotoxic effects, the most notable being heroin inhalation, known as “chasing the dragon” in adults [5,6].

Only a handful of pediatric patients with toxic leukoencephalopathy or malignant cerebellar edema due to opioids have been described in the literature, most due to accidental ingestion of prescribed opioids. The severity of this syndrome varies from mild with full clinical recovery as in our case to severe with profound cerebellar damage, hydrocephalus, tonsillar herniation, and death, as described in other case reports [7-21].

Cocaine exposure is common in children presenting to emergency rooms with neurological symptoms, including seizures, obtundation, delirium, dizziness, etc. [22]. Structural brain injury due to cocaine intoxication in children seems to be rare [22], and in adults mostly consists of strokes, intracranial hemorrhage, and cerebral ischemia [23]. The COVID-19 pandemic has caused many families to stay at home due to quarantine orders and the number of opioid and other toxic ingestions has increased [24].

We report the case of an eight-year-old child with concomitant drug ingestions, including fentanyl and cocaine, with clinical and radiologic findings of acute bilateral cerebellar leukoencephalopathy and signs of multi-organ hypoperfusion who completely recovered clinically within a few days.

## Case presentation

Our patient is an eight-year-old previously healthy child who was brought to medical attention to an outside hospital emergency department (ED) with altered mental status, obtundation, bradypnea, and miosis. The patient continued to deteriorate neurologically and exhibited agonal respiratory effort concerning acute respiratory failure. The child responded profoundly to empiric administration of 1mg naloxone IV with immediate improvement in mental status and respiratory effort raising suspicion of drug ingestion. Endotracheal intubation was deferred at that time. The patient was transferred to the ED of our institution, where they repeatedly became somnolent, bradypneic (with respiratory rates as low as 8 breaths per minute), tachycardic (with heart rates in the 150s), and hypoxic to the low 80s in room air, improving markedly with repeated naloxone boluses and subsequent naloxone infusion.

An initial workup included laboratory studies (Table 1), an EKG, a toxicology screen, and a head CT. A venous blood gas confirmed a combined respiratory and metabolic acidosis. Creatinine was elevated to 1.47mg/dL (0.20-0.73mg/dL), aspartate aminotransferase (AST) to 234U/L (10-40U/L), alanine transaminase (ALT) to 196 U/L (10-40U/L), lactic acid to 9mmol/L (0.3-1.9mmol/L), and troponin I to 0.61ng/mL (0.01-0.04ng/mL) raising concern for multi-organ effects. WBC was elevated to 34.2 x 10*3/uL (4.5-13.5 x 10*3) and glucose to 334mg/dL (70-99mg/dL) likely due to stress response. Electrolytes were mostly within normal ranges. The EKG showed sinus tachycardia with right bundle branch block and T-wave inversions in the precordial leads and prolonged QTc. Urine and serum toxicology screens were sent and resulted on hospital day 3.

**Table 1:** Laboratory values pH: potential of hydrogen; pCO2: partial pressure of carbon dioxide; HCO3: bicarbonate; BUN: blood urea nitrogen; AST: aspartate aminotransferase; ALT: alanine aminotransferase; WBC: white blood cells; n/a: not applicable

Test	Reference Ranges and Units	Hospital Day 1	Hospital Day 2	Hospital Day 5
Venous pH	7.31-7.41 pH	7.04	7.34	n/a
Venous pCO2	41-51 mmHg	78.1	43.5	n/a
Venous HCO3	23-28 mmol/L	21.2	23.2	n/a
Lactic acid	0.3-1.9 mmol/L	9	4.1	1.2
Sodium	135-145 mmol/L	137	141	141
Potassium	3.5-5.3 mmol/L	4.6	3.9	4.1
Chloride	97-110 mmol/L	99	109	107
BUN	7-23 mg/dL	12	13	11
Creatinine	0.2-0.73 mg/dL	1.47	0.73	0.4
Glucose	70-99 mg/dL	334	97	109
Calcium	8.7-10.7 mg/dL	8.3	8.7	9
AST	10-40 U/L	234	441	86
ALT	10-40 U/L	196	211	123
WBC (white blood cells)	4.5-13.5 x 10*3/uL	34.2	19.1	n/a
Hemoglobin	11.5-15.5 g/dL	11.3	12.8	n/a
Platelets	140-440 x 10*3/uL	312	277	n/a
Troponin I	0.01-0.04 ng/mL	0.61	6.03	0.27
Homocysteine	<11.4 umol/L	n/a	5.7	n/a
Rheumatoid factor	<14 IU/mL	n/a	< 14	n/a
Lipoprotein (a)	<75 nmol/L	n/a	14	n/a
Antithrombin III activity	80-120% normal	n/a	94	n/a
Factor V mutation	-	n/a	negative	n/a
Protein C, activity	70-180% normal	n/a	53	n/a
Protein S, activity	70-150% normal	n/a	32	n/a
Blood culture	-	No growth after five days	n/a	n/a
C-reactive protein	<10 mg/L	<1	n/a	n/a
Urine, fentanyl	<0.5 ng/mL	12	n/a	n/a
Urine, norfentantyl	<0.5 ng/mL	526	n/a	n/a
Urine, comprehensive drug screen	-	Caffeine; cocaine metabolite; diphenhydramine	n/a	n/a

**Figure 1:**
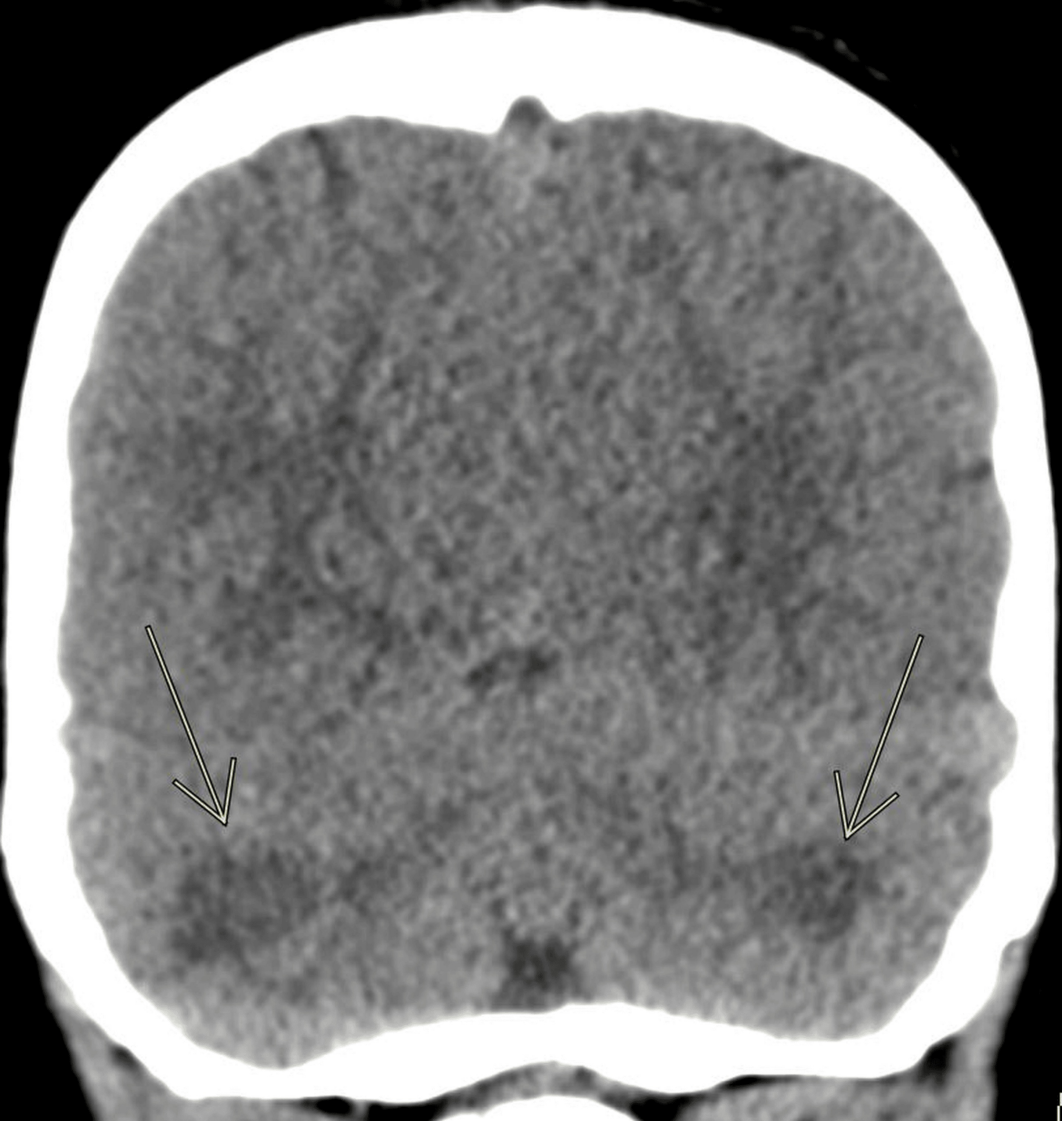
CT head Coronal CT obtained on the day of the presentation shows diffuse cerebellar white matter hypodensity.

Given the patient’s concerningly depressed mental status, a non-contrast head CT was obtained which showed diffuse cerebellar white matter hypodensities (Figure 1). Toxicology, social work, child protection medical team, pediatric neurology, and infectious disease were consulted. Our patient was admitted to the pediatric ICU for further monitoring and management.

The patient required a naloxone infusion for a total of 15 hours and never needed to be endotracheally intubated or treated with vasoactive agents, as they maintained adequate respiratory and hemodynamic function. The child continued to have confusion and altered mentation until about 36 hours after arrival to our hospital, but steadily improved clinically. AST and ALT peaked at 441U/L and 211U/L, respectively, and troponin I to 6.03ng/mL (0.01-0.04ng/mL) by the second hospital day, raising concern for multi-organ hypoperfusion or hypoxia at some point prior to their presentation. Fortunately, all laboratory abnormalities and organ functions improved or normalized throughout our patient’s hospital stay. Laboratory markers to exclude an infection or a hypercoagulable, inflammatory, and connective tissue disorder were obtained and were reassuring; these included serum homocysteine, rheumatoid factor, lipoprotein A, antithrombin III, factor V mutation, blood cultures, and C-reactive protein, and all were within normal limits or negative. Protein C/S levels were low and planned to be repeated as an outpatient. Our patient had an elevated WBC with neutrophil predominance that quickly normalized and a normal C-reactive protein. The need for a lumbar puncture was discussed but given their quick clinical improvement without antimicrobial treatment and lack of other infectious symptoms was not performed. Their EKG and QTc had normalized by hospital day 5.

**Figure 2:**
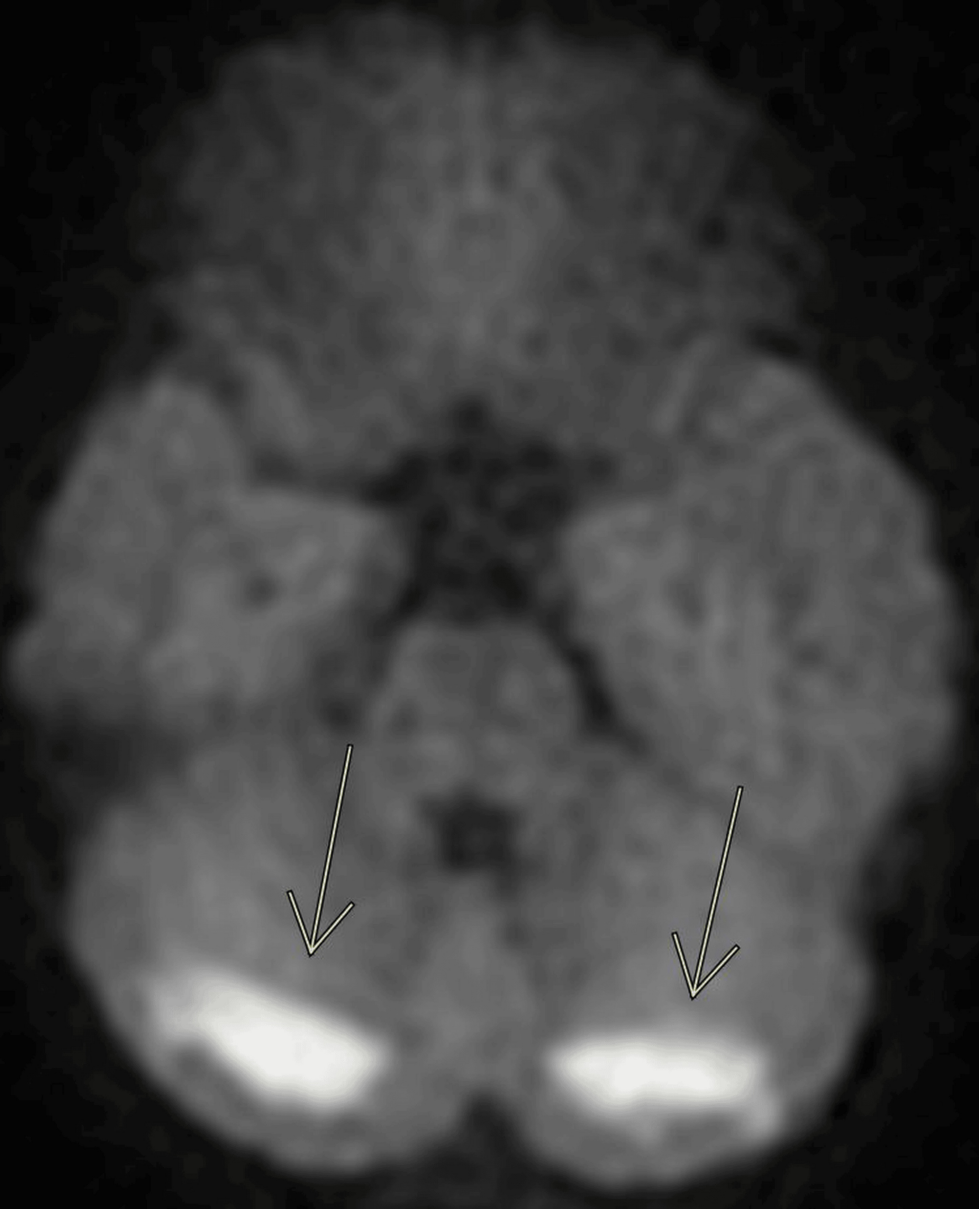
Brain MRI obtained on hospital day 3 Axial diffusion-weighted image (DWI) shows a bilateral hyperintense signal in the cerebellar white matter.

**Figure 3:**
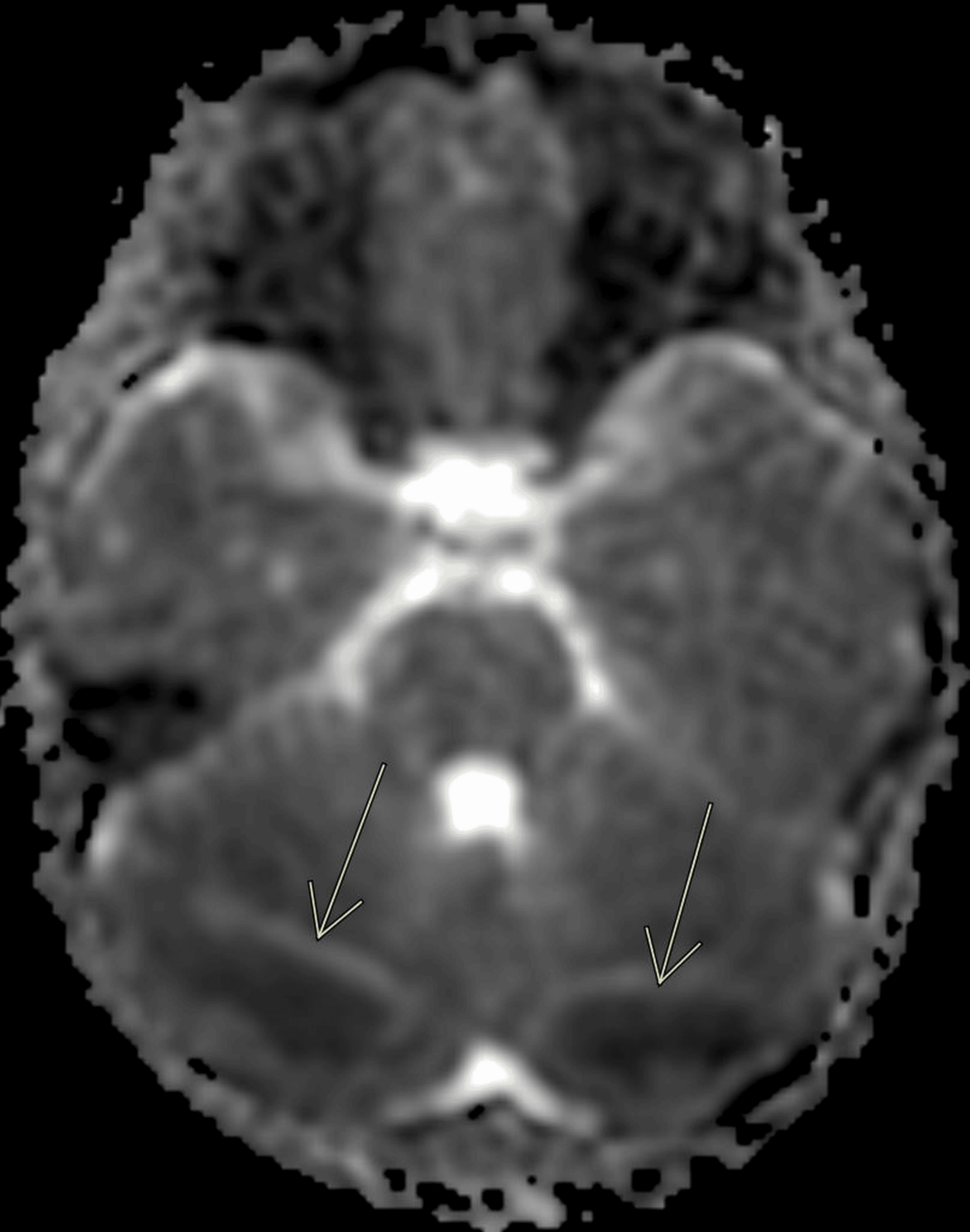
Brain MRI obtained on hospital day 3 Axial apparent diffusion coefficient (ADC) shows a bilateral hypointense signal in the cerebellar white matter, which confirms restricted diffusion.

**Figure 4:**
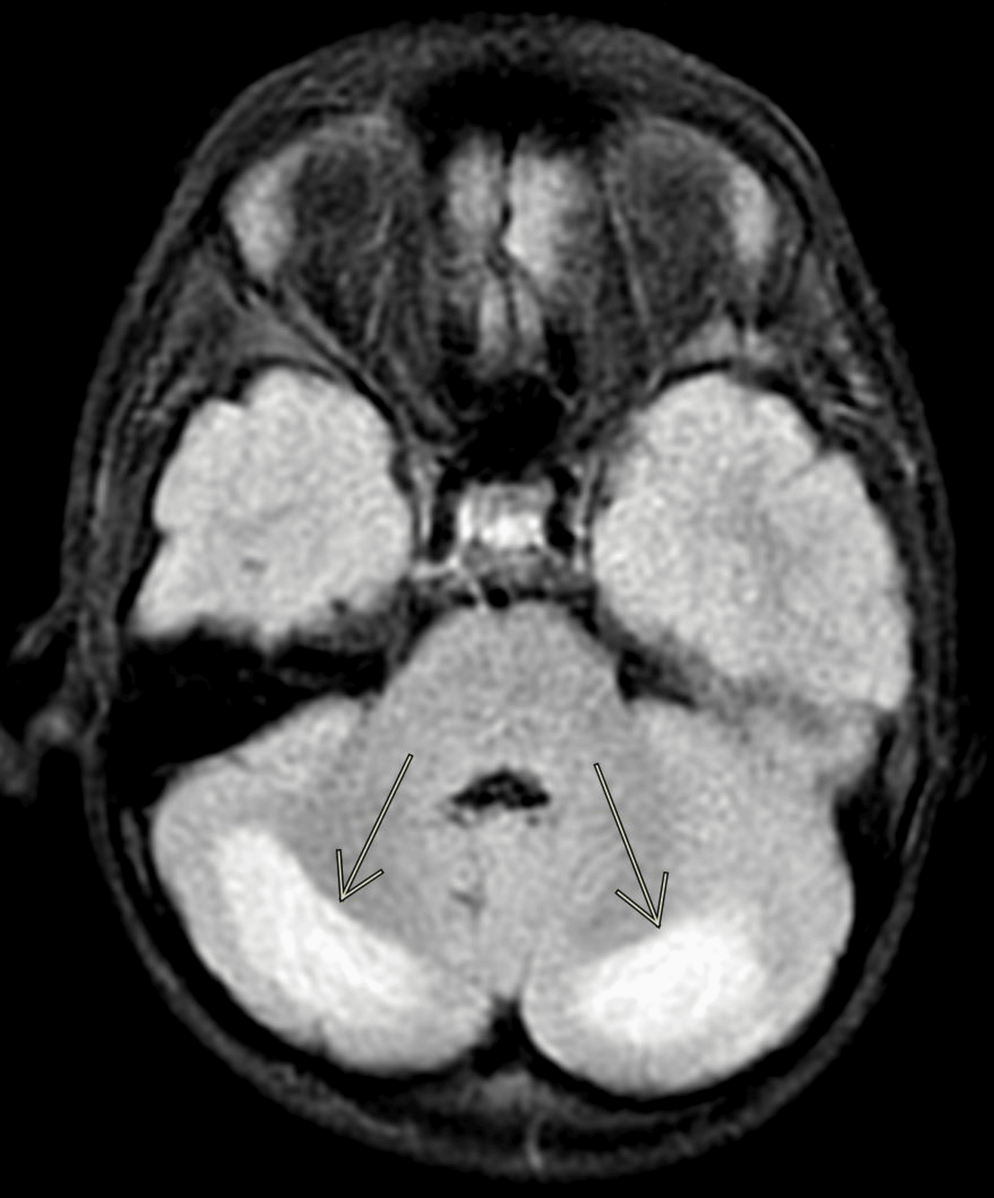
Brain MRI obtained on hospital day 3 Axial fluid-attenuated inversion recovery (FLAIR) shows a bilateral hyperintense signal in the cerebellar white matter.

A brain MRI was obtained on hospital day 3 to further delineate the cerebellar abnormalities seen on the head CT. The MRI was severely degraded by motion artifact, but did reveal bilateral symmetric white matter restricted diffusion and fluid-attenuated inversion recovery (FLAIR) hyperintensities in the posterior cerebellar hemispheres, with questionable subtle involvement of the bilateral occipital lobes (Figures 2-4). The brain MRI showed no neoplastic or demyelinating causes that would explain our patient’s condition.

Our patient underwent close neurologic monitoring. Their mental status slowly improved. Once able to communicate, our patient complained of right hip and thigh pain and numbness in the right leg. They were able to ambulate by hospital day 3, but their gait was unsteady and atactic. The patient had slightly decreased strength in the right lower extremity (4+/5), with normal strength in all other limbs. Deep tendon reflexes were 2+ in the upper extremities and 3+ in the bilateral lower extremities. No dysmetria of the upper extremities was noted on finger to nose test. The child endorsed mildly blurry vision until hospital day 3. By the time of discharge on hospital day 5, their neurologic exam had completely normalized and they seemed to have made a full recovery. Given our patient’s steady clinical improvement over a short period of time, repeat imaging was not obtained. Unfortunately, the child was lost to follow-up in our state and we do not have longer-term outcome data on them.

Our patient’s presentation with altered mental status, respiratory depression, miosis, and profound response to naloxone led to the ultimately correct assumption of a drug ingestion, most likely an opioid. The finalized toxicology report was not available until hospital day 3 and was positive for fentanyl (level of 12ng/mL), norfentanyl (level of 526ng/mL) (normal values <0.5ng/mL for both), cocaine, diphenhydramine, and caffeine. We do not have any known prior toxicology screening on our patient and therefore cannot say with certainty that they had exposure to these substances beyond the setting of this presentation.

Acute toxic cerebellar leukoencephalopathy is a clinicoradiologic diagnosis with both clinical symptoms and radiologic findings needed to provide this diagnosis. While our imaging findings have a broad differential, as do nearly all neuroimaging findings, in our case of suspected opiate use in a pediatric patient, the imaging findings were concordant with the clinical history and physical exam, thus allowing us to provide a most likely diagnosis of acute toxic cerebellar leukoencephalopathy.

We are uncertain how the patient was exposed to these substances. Additionally, screening and confirmatory toxicology testing cannot speculate as to chronic or past exposures to the patient. The patient’s presenting history was further confounded by the family members’ apparent altered mental status during the initial medical history, and further clarification was not possible due to social service involvement. Child protective services were involved early on in this case and ultimately took custody of our patient during their hospitalization due to high concern for abuse and/or neglect.

## Discussion

To our knowledge, this is the first report of a pediatric case of illicit fentanyl and cocaine ingestion causing acute cerebellar leukoencephalopathy. Despite severely depressed mental status on presentation and laboratory markers consistent with multi-organ hypoperfusion or hypoxia, our patient underwent a full clinical recovery within five days.

A handful of pediatric cases with acute cerebellar leukoencephalopathy have been described previously [7-13,15-21,25,26]. Most of the described cases have been attributed to accidental ingestions of prescribed opioids, such as methadone [11,13,15-17,19,20], morphine [12,18,25], oxycodone [25,26], buprenorphine [7], and fentanyl [8-10,21].

Neurologic outcomes with this condition seem to vary dramatically in severity from mild with full clinical recovery as in our case and as described in others [8,9,15,21], to severe with profound cerebellar damage, acute hydrocephalus, increased intracerebral pressure, cerebellar tonsillar herniation, and even death [8,10-12,19,26]. Neurosurgical interventions, in severe cases that progress to posterior fossa compressions, such as extraventricular drain placement, suboccipital craniectomy, and cerebellar resection have been shown to be potentially lifesaving with excellent neurological outcomes in some cases [10,13,15,18,25]. However, ideal management strategies, such as steroid administration, are yet to be defined.

Acute opioid-induced toxic leukoencephalopathy or malignant cerebellar edema seems to predominately affect the cerebellum in children [25], which might be explained by a difference in opioid receptor profiles in children versus adults [14]. Its pathologic mechanism is poorly understood and is thought to be due to a combination of hypoxia/ischemia and direct neurotoxicity. The cerebellum has a predominance of mu (μ) over delta receptors. Overstimulation of μ receptors with opioids may lead to a state of cellular energy depletion, which can be enhanced by the anoxia or hypoxia as a result of prolonged respiratory depression common in opioid overdose [27].

In adults who inhale heroin a similar syndrome (“chasing the dragon”) affecting the cerebral and cerebellum has been described, however, it differs in many ways in its presentation and usually occurs over prolonged exposure. Histologic examination of white matter collected from these cases shows spongiform degeneration, oligodendroglial vacuolization, and fluid entrapment between myelin lamellae, without demyelination [5,17]. Unfortunately, no histologic specimens of children with cerebellar leukoencephalopathy have been described in the literature to date.

In the few reports of children with opioid-induced cerebellar edema, non-contrast head CT revealed bilateral and symmetric cerebellar hypoattenuation, with hydrocephalus dependent on the magnitude of mass effect [8,10-12,15,17-19,25,26]. Further specific imaging via MR spectroscopy reveals results consistent with axonal injury without demyelination, with a lactate peak likely related to mitochondrial dysfunction [27].

Our patient’s drug screen noted the presence of cocaine, diphenhydramine, and caffeine in addition to fentanyl and norfentanyl. The degree to which these substances contributed to our patient’s symptomatology and imaging findings is unclear. Cocaine ingestion can cause neurologic manifestations in children such as seizures and mental status changes [22]; however, structural brain injury as seen in our patient has not been described that we are aware of.

We recommend children presenting with altered mental status or neurologic, particularly cerebellar symptoms, of unclear etiology, should undergo brain imaging as well as toxicology screening. Most children with suspected drug ingestions with neurologic or respiratory dysfunction will likely be monitored in an intensive care setting. Our patient steadily improved over their hospital stay which is consistent with the case of a toddler with accidental fentanyl ingestion recently reported [21]. However, several cases with acute toxic cerebellar leukoencephalopathy have been described in which the patients initially improved and then suddenly worsened two to three days after presentation due to worsening cerebellar edema, obstructive hydrocephalus, and cerebellar tonsillar herniation or death [8,10,25]. This can occur at a time after drug ingestion when most drugs would have likely been metabolized or cleared and less intense monitoring of the patient might be considered.

One case of severe toxic cerebellar leukoencephalopathy of a three-year-old girl is worth mentioning to also highlight the importance of awareness of this clinical entity. This patient who presented with mental status changes due to accidental morphine ingestion required sedation for mechanical ventilation which included fentanyl, as is common practice in many pediatric intensive care units (PICUs). Whether fentanyl administration contributed to her acute deterioration on hospital day 3, leading to worsening cerebellar edema, obstructive hydrocephalus, and emergent extraventricular drain placement is unclear [8].

## Conclusions

Awareness and prompt recognition of acute toxic leukoencephalopathy in children presenting with altered mental status or neurological symptoms of unclear etiology is essential to prevent deterioration and optimize treatment. Not all cases result in death or poor outcomes as several previously suggested case reports suggest, but some children can undergo a full clinical recovery without residual symptoms, as our patient did. However, given the potentially catastrophic outcome of acute toxic cerebellar leukoencephalopathy as a neurologic complication of opioid toxicity in addition to the better-known effect of respiratory depression, it is of utmost importance to raise awareness of this condition, especially since surgical intervention might be potentially life-saving. This is even more important during a time with increasing numbers of pediatric cases of opioid intoxication.
